# Anticoagulation Management in Patients with Pacemaker-Detected Atrial Fibrillation

**DOI:** 10.3889/oamjms.2016.053

**Published:** 2016-04-20

**Authors:** Lidija Poposka, Vladimir Boskov, Dejan Risteski, Jane Taleski, Ljubica Georgievska-Ismail

**Affiliations:** *University Clinic of Cardiology, Faculty of Medicine, Ss Cyril and Methodius University of Skopje, Skopje, Republic of Macedonia*

**Keywords:** atrial fibrillation, pacemaker, anticoagulants, thrombo-embolic events, asymptomatic

## Abstract

**INTRODUCTION:**

In patients with an implanted pacemaker, asymptomatic atrial fibrillation (AF) is associated with an increased risk of thrombo-embolic complications. There is still no consensus which duration of episodes of atrial fibrillation should be taken as an indicator for inclusion of oral anticoagulation therapy (OAC).

**MATERIAL AND METHODS:**

A total of 104 patients who had no AF episodes in the past and have an indication for permanent pacing were included in the study.

**RESULTS:**

During an average follow-up of 18 months, 33 of the patients developed episodes of AF. Inclusion of OAC was performed in 17 patients, in whom AF was recorded, although in all patients CHA2DS2-VASc score was ≥ 1. The inclusion of OAC showed a statistically significant correlation with increasing duration of episodes of AF (r = 0.502, p = 0.003). During the follow-up period none of the patients developed thrombo-embolic complication.

**CONCLUSION:**

Considering that our group of patients had no thrombo-embolic events, we could conclude that dividing the AF episodes in less than 1% in 24 hours and longer than 1% within 24 hours could be an indicator for decision-making to include OAK if the CHA2DS2-VASc score is ≥ 1.

## Introduction

Atrial fibrillation (AF) is the most common arrhythmia, whose incidence increases with age, and the prevalence in the general population is 1.5-2% [1]. AF is associated with twofold increase in mortality, threefold increase in the incidence of congestive heart failure and fivefold increase in the incidence of stroke [1, 2].

Oral anticoagulation (OAC) therapy significantly decreases the risk of stroke in all AF-patients [3, 4], but it increases the risk of bleeding. CHA_2_DS_2_ VASc score has been shown to be the best marker for detecting patients with very low risk for thrombo-embolic complications who do not benefit from OAC therapy [1].

Although AF is usually associated with palpitations, dyspnea, chest pain, anxiety, at least one third of patients with AF are completely asymptomatic. But the risk of stroke is equal whether AF is symptomatic or asymptomatic [5, 6].

One of the biggest challenges in this field is to identify patients with asymptomatic paroxysmal AF, and if indicated, that is, if CHA_2_DS_2_-VASc score is 1 or more than 1, to introduce OAC therapy.

AF can be very efficiently detected in patients with implanted pacemaker and the detection of atrial high rate episodes (AHRE) - defined as episodes of atrial frequency ≥220 bpm, lasting ≥5 minutes, has been proven to be an independent predictor of death or nonfatal stroke in these patients [7].

Previous studies have shown that in 90% of patients with implanted dual-chamber pacemakers, episodes of AF are asymptomatic, because of the lack of irregular ventricular contraction during arrhythmia [7, 8].

There is still no firm evidence on the duration of the AF-episode as a risk of thrombo-embolic complications and whether this risk increases with frequency, duration of a single episode or AF burden within a certain period of time. The optimal antithrombotic management in patients without atrial fibrillation, but with atrial high rate episodes, is not known.

The aim of this study was to evaluate the anticoagulation management in patients with atrial fibrillation detected from implanted pacemakers.

## Patients and Methods

A total of 104 patients with an indication for dual chamber pacemaker implantation were selected from the Clinic of Cardiology in the period from January 2013 to December 2014. Inclusion criteria in the study were no previous documented episodes of AF.

According to the indication and the decision of the cardiologist DDD, DDDR or VDD pacemaker was implanted on the non-dominant hand-side using standard procedure. The pacemaker was programmed in DDD, DDDR or VDD mode of stimulation with lower rate limit set at 60 bpm. No antiarrhythmic interventional algorithm was used. Atrial sensing was programmed at 30% of the measured P wave to avoid atrial over-sensing, or under-sensing. To prevent tracking fast atrial rhythms, mode switch was programmed at 175 bpm. Every pacemaker was programmed to record and show the maximum number of recorded episodes of high frequency atrial episodes.

Sensitivity and specificity for detection of atrial high rate episodes from the implanted devices was established in previous studies and it exceeds 95% [9].

Follow-up of patients was at one-month after implantation and every six months afterwards up to 24 months. At follow-up visits, patients were examined, pacemakers interrogated and therapies adjusted.

At each pacemaker interrogation the occurrence of atrial high rate episodes was monitored, their frequency and duration and mode-switch episodes. Patients with paroxysmal AF episodes were divided in two groups: patients who had episodes of AF <1% in 24h and patients who had episodes of AF >1% in 24h.

In patients who experienced persistent AF during the follow-up period, an attempt was made to restore sinus rhythm following anticoagulation according to the Guidelines for the management of atrial fibrillation [1, 2]. If this attempt was unsuccessful, permanent AF was accepted.

During the follow-up period every initiation of the OAC therapy was recorded, and it was made by the decision by an attending cardiologist. OAC therapy was either Vitamin K antagonist (acenocumarol) or non-vitamin K antagonist (NOAC).

### 

#### Statistical analysis

Categorical parameters were summarized as percentages and continuous parameters as mean ± SD. Comparisons between the two groups was performed using the Student’s t-test for continuous parameters and the Pearson’s chi-square test for categorical parameters. Assessment of correlation was done using the Spearman’s correlation analysis. Multiple logistic regression analysis was performed in stepwise order to determine independent predictors of OAC use.

All data analysis was performed using commercially available statistical programs and a p value ≤ 0.05 was considered significant.

## Results

Patients included in this study had a mean age of 66.3 ± 8.7 years, predominantly of male gender (62.5%).

Regarding pharmacological therapy, patients were treated mainly with angiotensin converting enzyme inhibitor (79 patients / 76.0 %), beta adrenergic blockers (26 patients / 25%), diuretics (46 patients / 54.2 %), acetyl- salicylic acid (48 patients / 46.2 %) and antiarrhythmics (8 patients / 7.2 %), of which 4 patients were treated with amiodarone and other 4 with calcium channel blockers. In 14 patients (13.5 %) during the follow up period beta blockers were added by the decision of the attending cardiologist.

During the average follow-up period of 18 months, from a total of 104 patients, 33 (31.7%) developed episodes of atrial fibrillation. Episodes with duration of <1% in 24 hours were observed in 20 (60.6%) patients, episodes > 1% in 24 hours were observed in 6 (18.2%) patients, persistent AF was diagnosed in 3 (9.1%) patients, and permanent AF was diagnosed in 4 (12.1%) patients. Spontaneous conversion to sinus rhythm occurred in 25 (75.8%) patients, drug conversion to sinus rhythm (with amiodarone) was conducted in 3 (9.1%) patients, and electrical cardio-version was performed in one (3.0%) patient.

In terms of symptoms, asymptomatic episodes of AF were observed in 22 (66.7%) patients and symptomatic episodes in 11 (33.3%) patients. The average time to detection of AF episodes was 1.39 ± 3.3 months from the beginning of the follow-up.

CHA2DS2-VASc risk score for susceptibility to the development of thrombo-embolic complications in all patients with AF was ≥1, which means oral anticoagulant therapy was indicated in all these patients, since the diagnosis of AF was made.

Despite that, OAC therapy was given to 17 patients only, out of 33 patients who experienced AF. OAC was given to 1 patient (3.0%) after 1 month follow-up, to 7 patients (21.2%) after 6 months follow-up, to 6 patients (18.2%) after 12 months follow-up, to 2 patients (6.0%) and to 1 patient (3.0%) after 24 months follow-up.

**Figure 1:**
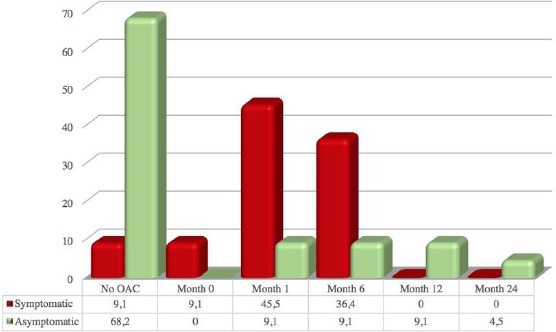
*Anticoagulation initiation in percentages during follow-up in months in patients with asymptomatic vs. symptomatic atrial fibrillation*.

A comparison of the initiation of OAC in patients with symptomatic or asymptomatic AF during the follow-up period showed a statistically significant difference between these two groups of patients (p = 0.006; Figure 1). Thus, a significantly larger percentage of patients with asymptomatic AF were not treated with OAC compared to patients with symptomatic AF (15 / 68.2% vs. 1 / 9.1%, respectively).

**Figure 2:**
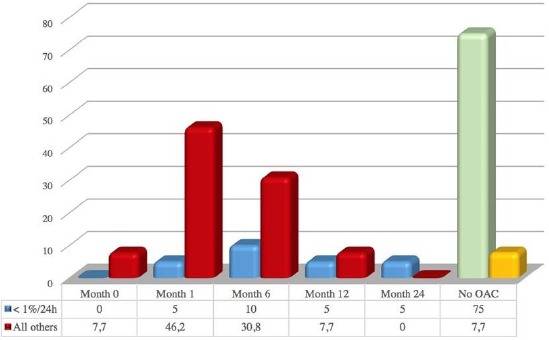
*Anticoagulation initiation in percentages during follow-up in months in patients with atrial fibrillation episodes with different duration (<1%/24h, vs. all others)*.

The inclusion of OAC therapy showed a statistically significant correlation with increasing length of duration of AF episodes (r = 0.502, p = 0.003). Regarding the timing of their involvement and the duration of AF, a statistically significant difference (p = 0.003) was observed between the group of patients with duration of <1% in 24 hours compared to all the other patients (Figure 2). OAC usually was not given to patients with short duration of AF episodes.

In order to see if there is a possible connection to the inclusion of OAC with certain demographic, clinical, and pacemaker features analysis of correlations was made and the results are presented in Table 1. Overall, these results indicate that the presence of CAD, diabetes, arterial hypertension (borderline significance), are significantly associated with the inclusion of OAC. Prolonged duration of AF and associated symptoms (symptomatic patients) are also significantly associated with the given OAC. Interestingly, administration of OAC was not statistically significantly associated with the value of CHA_2_DS_2_-VASc score.

**Table 1:** Correlation of OAC initiation with tested parameters

Parameters	Correlation
Coronary artery disease (%)	r = 0.366; p = 0.033

Diabetes mellitus (%)	r = 0.344; p = 0.046

Arterial hypertension (%)	r = 0.311; p = 0.074

Therapy with beta blockers (%)	r = 0.609; p = 0.0001

P wave duration on ECG (ms)	r = -0.387; p = 0.024

AF duration (<1%/24h *vs*. all others)	r = 0.658; p = 0.0001

AF (paroxysmal, persistent, permanent)	r = 0.661; p = 0.0001

Symptomatic AF (%)	r = 0.557; p = 0.001

Cumulative ventricular pacing	
Month 1	r = -0.398; p = 0.032
Month 6	r = -0.372; p = 0.056

Cumulative dual chamber pacing	
Month 1	r = -0.390; p = 0.037
Month 6	r = -0.349; p = 0.075

Searched AV on (%)	r = 0.625; p = 0.030

AF -atrial fibrillation; ECG – electrocardiography; AV -atrio-ventricular.

Variables presented in Table 1 were used for multivariate regression analysis in order to determine predictors for using OAC, and longer duration of AF (divided in <1% in 24 h and all others) has emerged as a significant independent predictor for inclusion of OAC (Table 2). It means that for every unit extension of the duration of the AF, decision to give OAC increases for 0.722 times (95 % CI 0.382-1.062, p = 0.0001).

**Table 2:** Multiple regression analysis for OAC initiation as a dependent variable from clinical features and duration of the AF as independent predictive variables

Coefficients^a^							

Model	Unstandardized Coefficients		Standar-dized Coeffi-cients	t	p	95% Confidence Interval for B	
	
	B	Std. Error	Beta			Lower Bound	Upper Bound
1- Constant - OAC <1, all others	-0.444 0.722	0.229 0.165	0.667	-1.994 4.382	0.064 0.000	-0.916 0.382	0.027 1.062

a) Dependent Variable: OAC yes, no

The assessment of the predictive value of the variables included in the model made with the Dubin-Watson’s test showed a value of 1.85, which confirmed that the variable is a truly independent predictor and is responsible for 66.7% of all reasons for inclusion of OAC in the therapy.

During the follow-up period none of the patients developed any thrombo-embolic complication.

## Discussion

According to the results of studies in patients with implanted devices AF daily burden is associated with an increased risk of ischemic stroke or transient ischemic attack [10, 11]. In our study during the follow-up period none of the patients has experienced ischemic stroke or transient ischemic attack, or any other thrombo-embolic complication. All patients who developed episodes of AF, according to CHA_2_DS_2_-VASc score which was ≥1, had an indication for OAC, but OAC was given only to 17 patients (51%) out of 33.

Patients with persistent and permanent AF (21% of all patients with AF) were mainly symptomatic, and OAC was given to all of them except one (Figure 2).

Patients with paroxysmal AF (79%) divided into two groups - patients with episodes of AF <1% in 24 hours (20 patients, or 60.6%) and patients with episodes of AF > 1% in 24h (6 patients, or 18.2%), showed a statistically significant correlation of OAC with increasing length of duration of AF episodes (r = 0.502, p = 0.003). OAC was not given to patients with short duration of AF episodes.

In the ASSERT study atrial high rate episodes, defined as episodes with heart rate above 190/min with a minimum duration of 6 minutes, increased the risk of thrombo-embolic complications (stroke or peripheral embolism) two times and also increased the risk of occurrence of clinically apparent AF [12]. Also, the higher risk of stroke was found in patients with higher CHADS VASc score. A question remains whether these short episodes of AF have direct connection with thrombus formation and further thrombo-embolic complications or simply indicate existence of changes in the atrial endothelium which are transient or persistent and increase the risk of stroke [13, 14].

Unlike the ASSERT study, the MOST study demonstrated a temporal relationship of AF episodes with stroke, but the duration of significant episodes of AF was defined as 5.5 h [7]. This was not confirmed in further studies. Although subclinical AF is associated with an increased risk of thrombo-embolic complications, very few patients had AF within the month before the acute thrombo-embolic event. This was presented in the Brambatti M *et al*. study, being an extension of the ASSERT study [15]. In other words, the temporal relationship between episodes of AF with subsequent thrombo-embolic complications needs to be clarified.

Capucci *et al*. found that in patients with dual-chamber pacemakers atrial high rate episodes longer than 5 minutes did not significantly increase thrombo-embolic risk, whereas episodes lasting longer than 24 h increased it (odds ratio 3.1) [16].

Notably on the other side, the results of the IMPACT trail showed that early initiation of OAC based on device-detected subclinical AF did not improve outcomes, in part because of temporal dissociation between AF and stroke, and possibly because of stroke mechanisms independent of AF [17]. The results of the IMPACT trial do not support urgent initiation of OAC and recommend decision for initiation of OAC based on individualized assessment of risk and benefit [17].

Nevertheless, we know that many of the patients with detected atrial high rate episodes develop clinical AF over time, and these episodes could be considered as a precursor of paroxysmal AF. Having in mind that OAC can produce bleeding complications there is still uncertainty about the optimal antithrombotic therapy in patients with subclinical AF.

In conclusion, the inclusion of OAC in patients with AF demonstrated a statistically significant correlation with the increasing duration of the episodes, with most OAC not given in patients with short duration of episodes (<1% in 24h).

Given that in our patients thrombo-embolic complications were not recorded, maybe the decision of the doctors to give or not OAC based on duration of the AF episode, with cut-off value longer or shorter than 1% in 24h was right, provided CHA2DS2-VASc score is ≥1.

Waiting for the results from the large trials addressing this issue - optimal antithrombotic therapy in patients with atrial high rate episodes: NOAH – AFNET 6 (Non-vitamin K antagonist oral anticoagulants in patients with atrial high rate episodes) and ARTESIA (Apixaban for the reduction of thrombo-embolism in patients with device-detected sub-clinical atrial fibrillation), we could follow the strategy to start with OAC if the detected atrial high rate episodes exceed >1% of the time in 24 h.
